# Associations between Dietary Fiber Intake in Infancy and Cardiometabolic Health at School Age: The Generation R Study

**DOI:** 10.3390/nu8090531

**Published:** 2016-08-30

**Authors:** Rafaëlle M. A. van Gijssel, Kim V. E. Braun, Jessica C. Kiefte-de Jong, Vincent W. V. Jaddoe, Oscar H. Franco, Trudy Voortman

**Affiliations:** 1 The Department of Epidemiology, Erasmus MC, University Medical Center, Office Na-2909, P.O. Box 2040, Rotterdam 3000 CA, The Netherlands; rmavgijssel@gmail.com (R.M.A.v.G.); k.braun@erasmusmc.nl (K.V.E.B.); j.c.kiefte-dejong@erasmusmc.nl (J.C.K.-d.J.); v.jaddoe@erasmusmc.nl (V.W.V.J.); o.franco@erasmusmc.nl (O.H.F.); 2 The Generation R Study Group, Erasmus MC, University Medical Center, Rotterdam 3000 CA, The Netherlands; 3 Department of Global Public Health, Leiden University, The Hague 3595 DG, The Netherlands; 4 The Department of Pediatrics, Erasmus MC, University Medical Center, Rotterdam 3000 CA, The Netherlands

**Keywords:** body fat, blood pressure, cohort, dietary fiber, early childhood, HDL-C, insulin, triglyceride

## Abstract

Dietary fiber (DF) intake may be beneficial for cardiometabolic health. However, whether this already occurs in early childhood is unclear. We investigated associations between DF intake in infancy and cardiometabolic health in childhood among 2032 children participating in a population-based cohort in The Netherlands. Information on DF intake at a median age of 12.9 months was collected using a food-frequency questionnaire. DF was adjusted for energy intake using the residual method. At age 6 years, body fat percentage, high-density lipoprotein (HDL)-cholesterol, insulin, triglycerides, and blood pressure were assessed and expressed in age- and sex-specific standard deviation scores (SDS). These five factors were combined into a cardiometabolic risk factor score. In models adjusted for several parental and child covariates, a higher DF intake was associated with a lower cardiometabolic risk factor score. When we examined individual cardiometabolic factors, we observed that a 1 g/day higher energy-adjusted DF intake was associated with 0.026 SDS higher HDL-cholesterol (95% CI 0.009, 0.042), and 0.020 SDS lower triglycerides (95% CI −0.037, −0.003), but not with body fat, insulin, or blood pressure. Results were similar for DF with and without adjustment for energy intake. Our findings suggest that higher DF intake in infancy may be associated with better cardiometabolic health in later childhood.

## 1. Introduction

Several studies suggest that dietary fiber (DF) is beneficial for various aspects of cardiometabolic health in adults, such as lower insulin and cholesterol concentrations, and a lower blood pressure [1,2]. High DF intake has been proposed to lower cardiometabolic risk through lower absorption of cholesterol and fat, improved glucose and insulin metabolism after meals, or via increased satiety and a subsequent lower energy intake [3]. However, many of the cardiometabolic health consequences in adulthood are preceded by abnormalities that might begin in childhood [4]. For example, overweight often already occurs in early childhood and is associated with a higher risk of overweight, type 2 diabetes, hypertension, dyslipidemia, and atherosclerosis in later life [4,5]. Small changes in other cardiometabolic risk factors can also already start during childhood and predict later cardiometabolic disease risk [6,7]. Therefore, it is important to focus on cardiometabolic health and its determinants already in childhood. A few previous studies in children suggested that the beneficial effect of DF on cardiometabolic health may already be present in childhood: a higher DF intake was, for example, associated with a lower body fat percentage in children around the age of 9 years [8], and with lower serum total cholesterol in children at ages of 13 months to 9 years [9].

However, these previous studies on DF intake in children in relation to body composition and metabolic risk factors focused on school-age children and adolescents [8,9,10,11,12,13,14,15,16], whereas diet might be important for cardiometabolic health already earlier in childhood [17]. Whether DF intake in early childhood is associated with cardiometabolic risk remains unknown and there is a lack of well-founded guidelines for adequate DF intake in young children [18,19,20]. Potential effects of DF intake might also differ by type of DF, such as soluble versus insoluble DF [3,21,22,23,24]. An observational study in adolescents showed that a higher intake of specifically soluble DF was associated with a reduction of visceral body fat over a period of 2 years [13]. However, data about the effects of different sources of DF is scarce [3].

Therefore, we investigated the association between DF intake in infancy and cardiometabolic health at the age of 6 years in a large prospective cohort. Additionally, we examined whether associations were explained by differences in energy intake and we explored whether associations differ for DF from different food sources.

## 2. Subjects and Methods

### 2.1. Study Design and Subjects

In this study we examined data from The Generation R Study, a population-based prospective cohort study from fetal life onward in Rotterdam, The Netherlands [25]. Pregnant women were enrolled between April 2002 and January 2006, and 7893 live-born children were available for postnatal follow-up, of whom 4215 had a Dutch ethnic background [25].

**Figure 1:**
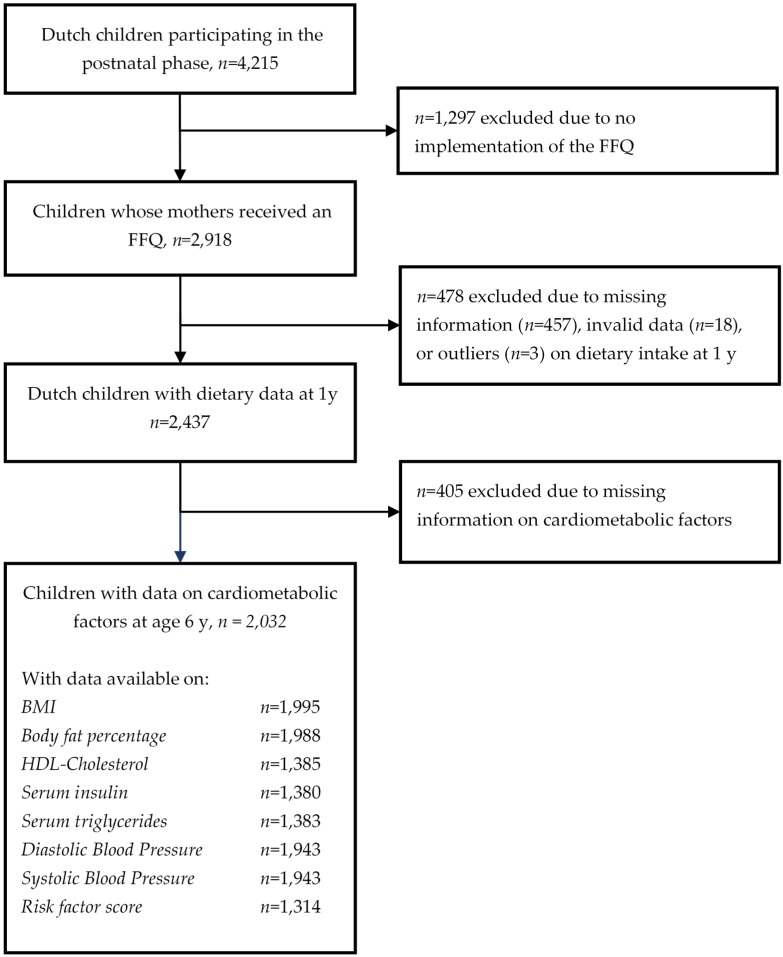
Flow chart of study participants included in the analysis.

Because the food-frequency questionnaire (FFQ) was designed for dietary assessment of a Dutch population and was validated in Dutch children, we restricted our analyses to Dutch children. Children without information on diet (*n* = 1778) or any of the cardiometabolic health measurements (*n* = 405) were excluded, resulting in a study population of 2032 children (Figure 1). Because not all of these children had information available on all outcomes, the population for analysis ranged from 1314 to 1995 per cardiometabolic outcome. The study was approved by the local Medical Ethics Committee of Erasmus Medical Center, Rotterdam (MEC 198.782/2001/31, 2001), and written consent was given by parents.

### 2.2. Dietary Intake Assessment

Dietary data were collected using a 211-item semiquantitative FFQ, as described in detail elsewhere [26,27]. This FFQ was validated against three 24 h-recalls and the intraclass correlation coefficient was 0.7 for DF intake [27]. The median age of the children was 12.9 (interquartile range (IQR) 12.6–13.9) months. Total DF intake was calculated using the Dutch Food Composition Table (NEVO), where DF is defined as plant cell wall components that are not digestible by human digestive enzymes, including for example, lignin, cellulose, hemicellulose, and pectin [28]. Thereafter, we divided DF intake in DF from four different food groups, based on the different types of DF that are mainly present in these food groups [3,22]: (1) DF from cereals, as proxy for insoluble DF, defined as fiber from bread, cereals, pasta, rice, cookies, cakes, pastries, crackers, and ready-to-eat meals; (2) DF from potatoes and potato products, containing resistant starch; (3) DF from fruits and vegetables; and (4) DF from legumes, containing different types of soluble DF, insoluble DF, and resistant starch [29].

### 2.3. Cardiometabolic Health Assessment

Children’s height and weight up to the age of 4 years were repeatedly measured during routine visits to Child Health Centers [30]. At a median age of 5.9 (IQR 5.8–6.1) years, children visited our dedicated research center in the Sophia Children’s Hospital in Rotterdam where they were examined in detail. As a primary outcome we used a cardiometabolic risk factor score including the components body fat percentage (BF%), high-density lipoprotein cholesterol (HDL-C), insulin, and triglyceride concentrations, and diastolic blood pressure (DBP) and systolic blood pressure (SBP). Body weight, measured with a mechanical personal scale (SECA), and body height, without shoes and heavy clothes, were measured to the nearest 0.1 kg or 0.1 cm, respectively, and BMI was calculated (kg/m^2^). Body fat was determined using a dual-energy X-ray absorptiometry scanner (iDXA; General Electrics-Lunar, 2008, Madison, WI, USA) and enCORE software v.13.6 (GE Healthcare, Little Chalfont, UK) [31]. Total body fat was expressed as percentage of total body weight to define BF%. Additionally, we calculated fat mass index (FMI) as fat mass (kg)/m^2^.

Non-fasting blood samples were drawn for measurements of HDL-C, insulin, and triglyceride concentrations, using enzymatic methods (using a Cobas 8000 analyzer, Roche, Almere, The Netherlands) [32]. Quality control samples demonstrated intra-assay and inter-assay coefficients of variation ranging from 0.77% to 1.69%. DBP and SBP were measured, while the children were lying down, at the right brachial artery, using the validated automatic sphygmomanometer Datascope Accutorr Plus™ (Paramus, NJ, USA) [33]. Measurements were repeated four times with 1 min intervals and we used the mean of DBP and the mean of SBP of the last three measurements.

For all outcomes we calculated age- and sex-specific standard deviation scores (SDS) on the basis of our study population. Outliers, defined as the SDS ≥ |3.29| [34], were excluded (*n* = 0–7 per outcome). Individual cardiometabolic outcomes were combined into a continuous cardiometabolic risk factor score as described elsewhere [17,35]. This score included the sum of age- and sex-specific SD scores of BF%, the inverse of HDL-C, insulin, triglycerides, SBP, and DBP and was transformed into an SD score. A higher score is indicative of a less favorable cardiometabolic profile.

### 2.4. Covariates

Maternal age, household income, educational levels of both parents, and proxies for mother’s pre-pregnancy cardiometabolic health (i.e., hypercholesterolemia, diabetes mellitus, or hypertension) were obtained with a questionnaire at enrolment in the study. Educational level of both parents was assessed with the same questionnaire and highest finished education was categorized into: no higher education; one parent with higher education; or both parents finished higher education, with higher education defined as higher vocational training, a Bachelor’s degree, or university degree [36].

Maternal height and weight were measured at the research center at enrollment in the study, and BMI was calculated. Information about smoking, alcohol intake, and folic acid supplementation during pregnancy, as markers of health-conscious behavior of the mother, was obtained with questionnaires during pregnancy [27]. Information about pregnancy complications (i.e., gestational hypertension, preeclampsia, or gestational diabetes mellitus) was retrieved from medical records.

Medical records and hospital registries were used to collect information about child’s sex, birth weight and gestational age. Sex- and gestational age-specific *z*-scores for birth weight were calculated using reference data [37]. Information about receiving breastfeeding at 4 months and timing of introduction of fruit and vegetables was collected with postnatal questionnaires [27].

A food-based diet score for preschool children was used to assess overall diet quality [27] and macronutrient intakes and glycemic load of the diet were calculated [38] from data obtained with the FFQ. Information about receiving dietary supplements (e.g., vitamins and mineral supplements) as a proxy of health-conscious behavior, was retrieved using the FFQ. Information about average screen time as proxy for sedentary behavior and about time spent walking or bicycling to school and playing outside as proxies for physical activity, and smoking in the household (categorized into never; less than once per week; or once per week or more) was obtained with a questionnaire at the child’s age of 6 years.

### 2.5. Statistical Analysis

To explore whether potential associations of DF intake with cardiometabolic health were explained by energy intake, we examined both absolute DF (i.e., not adjusted for energy intake) and energy-adjusted DF intake. DF was adjusted for energy intake using the residual method [39]. Insulin was root-transformed, to obtain a normal distribution. Potential nonlinearity of the associations was assessed using natural cubic spline models with two to four degrees of freedom [40]. Because there were no indications for nonlinear associations for any of the outcomes (all *p* > 0.05), we assessed all associations using linear regression analyses only. In these models, we analyzed total DF intake and DF intake from different sources (per 1 g/day) and the cardiometabolic risk factor score and individual cardiometabolic health components at the age of 6 years.

In the crude model, we included child’s age at FFQ and sex. To identify potential dietary confounders, we first examined correlations of DF intake with intake of total protein, vegetable protein, animal protein, fat, polyunsaturated fatty acids, monounsaturated fatty acids, saturated fatty acids, carbohydrates, glycemic load, and the diet quality score. We observed a strong correlation for energy intake, glycemic load, and the diet score with DF intakes, and therefore included these variables in the analyses. To avoid overadjustment, the diet quality score and glycemic load were adjusted for DF intake using the residual method. All other covariates described in the Covariates section were included as potential confounders using the manual forward stepwise method starting with the crude model. Variables were retained in the covariate-adjusted model when they resulted in a ≥5% change of the effect estimates of absolute or energy-adjusted DF intake on at least one of the outcomes. Following this procedure, all variables were retained, so covariate-adjusted models were adjusted for: maternal age, maternal BMI, household income, educational level of the parents, smoking, alcohol intake and folic acid supplementation during pregnancy, pregnancy complications, child’s birth weight, sex, receiving breastfeeding at 4 months, timing of introduction of fruit and vegetables, age at FFQ, receiving dietary supplements, glycemic load, diet quality score, physical activity, screen time, and smoking in the household.

We tested for potential effect modification by child’s sex, age at FFQ, and overweight status at the age of 6 years in the crude and covariate-adjusted models. We additionally explored whether DF intake was associated with repeatedly measured height, weight, and BMI using multivariable linear mixed models including the same covariates as in the main models. For the cardiometabolic risk factor score, we performed sensitivity analyses in which we excluded individual components from the score one at a time. Furthermore, we repeated the analyses in a selection of the children with complete data on all cardiometabolic outcomes (*n* = 1314).

Missing values of covariates were multiply imputed (*n* = 10 imputations) according to the Fully Conditional Specification method (predictive mean matching), with the assumption of no monotone missing pattern [41]. Effect estimates and population characteristics (Supplementary Materials Table S1) were similar before and after imputation and we report the pooled results after the multiple imputation procedure. Statistical analyses were performed using SPSS 21.0 (IBM Corp., version 21.0, Armonk, NY, USA) and R (The R Foundation for Statistical Computing, version 3.2.0, Aalborg, Denmark) and results were considered statistically significant at a *p*-value < 0.05.

## 3. Results

### 3.1. Population Characteristics

The population characteristics are shown in Table 1. At the age of 1 year, mean ± SD intake of total DF was 15.0 ± 4.3 g/day, with a range of 3.0–38.6 g/day, with 46.4% of the children having a DF intake above the Dutch recommended intake of 15 g/day for 1–3 year old children [42]. Most of the DF in the diet of our study population came from cereal products (median (IQR) 8.0 (6.2–10.0) g/day) and from fruits and vegetables (4.7 (3.2–6.2) g/day). Overall, parents of most of the children had a high educational level and a high income.

### 3.2. Associations between DF Intake and Cardiometabolic Health

Table 2 presents the crude and covariate-adjusted associations between DF intake and energy-adjusted DF intake and age- and sex-adjusted outcomes of cardiometabolic health. In covariate-adjusted models, a 1 g/day higher energy-adjusted DF intake was associated with a 0.022 (95% CI −0.038, −0.006) SD lower cardiometabolic risk factor score. The effect estimate for DF without adjustment for energy was similar (−0.022 SDS; 95% CI −0.037, −0.006).

Thereafter, we analyzed the components of the cardiometabolic risk factor score separately (Table 2). A 1 g/day higher energy-adjusted DF intake was associated with 0.026 SDS (95% CI 0.089, 0.042) higher HDL-C concentrations. In addition, a higher energy-adjusted DF intake were associated with 0.020 SDS lower triglyceride concentrations (95% CI −0.037, −0.003). DF intake was not associated with BF%, insulin, DBP, or SBP. Intake of DF was also not associated with repeatedly measured height (−0.004 (95% CI −0.012, 0.005)), weight (−0.007 (95% CI −0.017, 0.003)), or BMI (−0.001 (95% CI −0.010, 0.008)), or with FMI at 6 years (−0.003 (95% CI −0.009, 0.009)). Also for the individual cardiometabolic outcomes, effect estimates with and without energy adjustment were similar (Table 2).

### 3.3. Associations between DF Intake from Different Sources and Cardiometabolic Health

Table 3 presents the covariate-adjusted associations between intake of DF from different sources and cardiometabolic health. A 1 g/day higher energy-adjusted DF intake from potatoes was associated with a 0.051 SDS (95% CI −0.094, −0.009) lower cardiometabolic risk factor score, and a 0.076 SDS lower triglyceride concentration (95% CI −0.121, −0.031). Effect estimates without energy adjustment were similar and in addition, there was a positive association between DF intake from potatoes and HDL-C (0.028 SDS; 95% CI 0.016, 0.072).

We observed that a 1 g/day higher energy-adjusted DF intake from fruit and vegetables was associated with a 0.028 SDS (95% CI 0.001, 0.054) higher HDL-C (similar without energy adjustment). Effect estimates for DF from legumes were similar to those obtained for total DF, however, the confidence intervals were wide and none of the associations for DF from legumes was statistically significant. For DF intake from cereals, we observed no associations with any of the outcomes.

### 3.4. Additional Analyses

We observed no significant interaction of DF intake with child’s sex or age at FFQ on any of the outcomes. Crude associations were comparable with those after adjustment for confounders (Supplementary Materials Table S2). When we restricted our analyses to children with complete data on all cardiometabolic factors, we observed similar results as obtained for the full sample (Supplementary Materials Table S3). When we analyzed the cardiometabolic risk factor score without triglycerides (TG) or without HDL-C in sensitivity analyses, effect estimates only slightly attenuated (Supplementary Materials Table S4). Finally, we observed no significant differences in DF intake, BF%, or blood pressure between children with or without blood samples (Supplementary Materials Table S5).

## 4. Discussion

This is the first study that investigated the association between DF intake, including DF from different sources, and cardiometabolic health in a large cohort of young children. Overall, a higher DF intake was associated with a lower cardiometabolic risk factor score. This was mainly determined by a better blood lipid profile. Furthermore, we observed that the association between DF intake and better cardiometabolic health was not explained by differences in energy intake and seemed to be primarily driven by intake of DF from potatoes, fruits, and vegetables. Although effect sizes were modest and without clinical implications on the individual level, small variations in cardiometabolic risk factors in childhood have been shown to predict health in later life [6,7], and a high DF intake already in early childhood may thereby contribute to a reduction in the cardiometabolic disease burden of a population.

Our observation that a higher DF intake was associated with better combined cardiometabolic health is in line with previous studies in adolescents and adults that showed that a higher DF intake was associated with a lower risk of metabolic syndrome [1,43]. Although associations with all individual cardiometabolic outcomes were in the expected direction, we observed that the association with overall cardiometabolic health in our population was mainly driven by higher HDL-C and lower triglyceride concentrations. In line with our results, Ludwig et al. [2] showed that a higher DF intake was associated with higher HDL-C and with lower triglyceride concentrations in adults. In contrast, two previous studies that examined these associations in children found no associations between DF intake and HDL-C or triglycerides [9,16]. However, these studies were small (*n* = 543 and *n* = 147) and may have been underpowered to detect such associations. A potential beneficial effect of DF intake on blood lipid levels could be explained by two mechanisms. First, DF decreases gastric emptying, which reduces the acute postprandial responses, and thereby controls glucose and lipid concentrations after a meal [24,44]. In the long-term, lower fluctuations of glucose and lipids can reduce the risk of cardiometabolic disorders. Second, absorption of lipid is affected by dietary fiber intake, which leads to a reduction in hepatic cholesterol. [45].

Although associations were all in the expected direction (i.e., improved cardiometabolic health), DF intake was not significantly associated with the individual cardiometabolic components BF%, insulin, DBP, or SBP in our population. Results from previous studies in children and adolescents are mixed, with studies reporting either null or beneficial associations for higher DF intake. Jenner et al. [15] reported associations between a higher DF intake and energy-adjusted DF intake with a lower DBP in boys, but not in girls, at the age of 9 years. Two studies observed no associations of DF with BF% in young children [11] or with BF% and insulin dynamics in adolescents [12], whereas three other studies reported that higher energy-adjusted DF intake was associated with lower BMI [8,10], lower visceral adiposity [13], and improved insulin-related outcomes [10] in children and adolescents. Since most of these studies were performed in older children [8,10,13] or in higher risk populations, such as obese teenagers [10,13], there may have been insufficient variability in our young and generally healthy population to observe a potential beneficial effect of DF intake on these individual cardiometabolic factors. Although very high DF intake has been hypothesized to have harmful effects on growth, studies do not support that high DF intake compromises growth of children in developed countries [18,20]. In line with this, we observed no associations of DF intake with repeatedly measured height or weight and we observed no indications for nonlinear associations of DF intake with any of the growth or cardiometabolic factors, suggesting that associations did not differ for low or high DF intakes in our study population with a generally high DF intake.

Considering that DF intake may result in satiation that could lead to a lower energy intake [24], associations between DF intake and cardiometabolic health were hypothesized to be explained by differences in energy intake. However, we observed no clear differences in associations for DF with or without energy adjustment. If any, the effect estimates became stronger after adjustment for energy intake, which is in line with results from two previous studies among children that showed larger effect estimates of DF on cardiometabolic outcomes after adjustment for energy [9,15]. This suggests that the effects of satiation may not play a major role among young children. In other words, a high DF intake does not necessarily displace energy intake [9] and a higher DF intake might be beneficial for cardiometabolic health via other mechanisms.

These other mechanisms may differ for different types and food sources of DF. In our study, we observed that associations of DF intake with blood lipids were mainly explained by intake of DF from fruits, vegetables, and potatoes, but not from cereals. Fruits and vegetables contain hemicellulose A, pectin, gums, and mucilage; and these types of DF may increase the viscosity that result in distension of the stomach [22]. Potatoes contain resistant starch, and in line with our results on blood lipids, Higgins et al. [23] reported that a replacement of 5.4% of dietary carbohydrates with resistant starch increased postprandial lipid oxidation. Resistant starch has also been associated with an increase in satiety in adults [46], and the mechanism behind lowering triglyceride concentrations probably involves increased intestinal viscosity [47]. Other studies showed that the solubility of DF could influence the associations [13,14]. Unfortunately, we were only able to make a division of DF by food sources, and not by type of DF. Therefore, there is still an overlap between intake of soluble and insoluble DF in our analyses. Finally, because of the low legume intake in our population, we might not have been able to detect associations for DF intake from legumes.

Our study was performed in a large population-based cohort with information available on many potential confounders. Although not all information on confounders was complete for all participants, we used multiple imputation of covariates to reduce attrition bias. Another strength is the availability of blood samples to measure detailed markers of cardiometabolic health. Blood samples were available in only 68.3% of the children, which may have led to selection bias. However, we observed no significant differences in DF intake, BF%, and blood pressure between children with or without blood samples [17] and results were similar when we restricted our analyses to the subjects with complete data on cardiometabolic health available, suggesting that bias due to missing blood samples may not be a large issue in the current analyses. A limitation is that blood lipids and insulin concentrations were measured in blood samples that were collected in non-fasting state. According to studies in adults, fasting time has little influence on cholesterol levels [48], but concentrations of triacylglycerol [48] and insulin [49] vary more substantially with differences in fasting time. Assuming that fasting time of the children when visiting our research center is randomly distributed, this measurement error would have led to non-differential misclassification of the outcomes and may therefore have resulted in an underestimation of our effect estimates for associations with insulin and blood lipids.

Regarding the nutritional assessment, an FFQ is an appropriate method because it reflects habitual diet. Furthermore, although FFQs are prone to measurement error, our validation study showed an intra-class correlation coefficient of 0.7 for DF intake [26,27], indicating a reasonably good validity. Unfortunately, we had no information available on intake of specific subtypes of DF, such as soluble and insoluble DF. A limitation is that this is an observational study, therefore, we cannot establish a causal relation. Although we had information available on many potential confounders, residual confounding from, for example, physical activity may still be an issue. Finally, we did not have cardiometabolic health data at the age of 1 year or dietary data at the age of 6 years, consequently, we were not able to perform longitudinal analyses for the cardiometabolic factors.

## 5. Conclusions

Results of this prospective cohort study suggest that a higher DF intake in infancy is associated with better combined cardiometabolic health, especially with a healthier blood lipid profile. These associations were not explained by differences in energy intake. Intake of DF from potatoes, fruits, and vegetables rather than cereals seemed to drive this association. Future studies should investigate the longitudinal association of DF intake, including different subtypes of DF, in early childhood with long-term cardiometabolic health.

## Figures and Tables

**Table 1:** Population characteristics (*n* = 2032).

	Mean ± SD, Median (IQR), or *n* (%)
**Infancy Characteristics**	
Gestational age at birth (weeks)	40.1 (39.3–41.1)
Birth weight (g)	3499 ± 563
Girls (*n*)	1031 (50.7%)
Receiving breastfeeding	
● Never	272 (13.3%)
● Partial in the first 4 months	1154 (56.8%)
● Exclusively in the first 4 months	606 (29.9%)
Timing of introduction of fruits and vegetables	
● <4 months	162 (7.9%)
● 4–6 months	1716 (84.4%)
● ≥6 months	154 (7.7%)
**Characteristics at Dietary Assessment**	
Age (months)	12.9 (12.6–13.9)
Dietary fiber (DF) intake (g/day)	15.0 ± 4.3
● DF from cereals	8.0 (6.2–10.0)
● DF from potatoes	1.1 (0.4–1.9)
● DF from fruit and vegetables	4.7 (3.2–6.2)
● DF from legumes	0.2 (0.0–0.6)
Energy intake (kcal/day)	1267 (1070–1491)
Receiving any dietary supplements	973 (47.9%)
**Characteristics at Cardiometabolic Health Assessment**	
Age (year)	5.9 (5.8–6.1)
Height (cm)	118 (115–122)
Weight (kg)	21.8 (20.2–23.8)
BMI (kg/m^2^) (*n* = 1995)	15.6 (15.0–16.5)
Body fat percentage (*n* = 1988)	23.1 (20.4–26.4)
Serum HDL-cholesterol (mmol/L) (*n* = 1385)	1.33 ± 0.30
Serum insulin (pmol/L) (*n* = 1380)	114 (63.8–183.6)
Serum triglycerides (mmol/L) (*n* = 1383)	0.98 (0.72–1.29)
Diastolic blood pressure (mmHg) (*n* = 1943)	60 ± 6
Systolic blood pressure (mmHg) (*n* = 1943)	102 ± 8
Physical activity (h/day)	1.60 (1.00–2.43)
Screen time (h/day)	1.14 (0.75–1.71)
Seldom or no smoking in household	1829 (90.0%)
**Parental Characteristics**	
Maternal age at enrolment (year)	32.3 (29.7–34.6)
Maternal BMI at enrolment (kg/m^2^)	23.3 (21.7–25.8)
Household income ≥ €2,200 per month	1586 (78.0%)
Educational level parents	
● No higher education	421 (20.7%)
● One parent higher education	495 (24.4%)
● Both parents higher education	1116 (54.9%)
Maternal hypercholesterolemia, diabetes mellitus, or hypertension	93 (4.6%)
Smoking during pregnancy	
● Never	1606 (79.0%)
● Until pregnancy was known	213 (10.5%)
● Continued	213 (10.5%)
Alcohol consumption during pregnancy	
● Never	627 (30.9%)
● Until pregnancy was known	364 (17.9%)
● Continued	1041 (51.2%)
Use of folic acid supplements during pregnancy	
● Start periconceptional	1245 (61.3%)
● Start in first 10 weeks of pregnancy	599 (29.4%)
● No	188 (9.3%)
Gestational hypertension, preeclampsia, or gestational diabetes mellitus	183 (9.1%)

Abbreviations: DF, dietary fiber; HDL, high density lipoprotein; IQR, interquartile range.

**Table 2:** Crude and covariate-adjusted associations between DF intake and energy-adjusted DF intake and cardiometabolic outcomes.

	DF Intake (per 1 g/day)		Energy-Adjusted DF Intake (per 1 g/day) ^1^	
	Crude Model ^2^	Covariate-Adjusted Model ^3^	Crude Model ^2^	Covariate-Adjusted Model ^3^
Cardiometabolic risk factor score	−0.014 *	−0.022 *	−0.023 *	−0.022 *
*n* = 1314	(−0.024, −0.003)	(−0.037, −0.006)	(−0.038, −0.007)	(−0.038, −0.006)
BF% (SDS)	−0.005	−0.005	−0.004	−0.003
*n* = 1988	(−0.014, 0.004)	(−0.017, 0.007)	(−0.016, 0.008)	(−0.015, 0.010)
HDL-C (SDS)	0.018 *	0.027 *	0.023 *	0.026 *^,4^
*n* = 1385	(0.012, 0.024)	(0.011, 0.044)	(0.007, 0.039)	(0.009, 0.043)
Insulin (SDS)	0.001	0.002	−0.005	−0.003
*n* = 1380	(−0.005, 0.007)	(−0.014, 0.019)	(−0.013, 0.003)	(−0.020, 0.015)
Triglycerides (SDS)	−0.008	−0.019 *	−0.015	−0.018 *^,4^
*n* = 1383	(−0.020, 0.004)	(−0.035, −0.003)	(−0.031, 0.001)	(−0.036, −0.002)
DBP (SDS)	−0.006	−0.006	−0.004	−0.003
*n* = 1943	(−0.015, 0.004)	(−0.019, 0.008)	(−0.017, 0.009)	(−0.017, 0.011)
SBP (SDS)	−0.001	−0.007	−0.009	−0.009
*n* = 1943	(−0.006, 0.004)	(−0.020, 0.007)	(−0.022, 0.005)	(−0.023, 0.005)

Values are based on multivariable linear regression models and reflect differences (95% confidence interval) in individual cardiometabolic outcomes and in cardiometabolic risk factor score (age- and sex-adjusted SDS) per 1 g/day increase in DF intake. ^1^ DF was analyzed as energy-adjusted DF using the residual method and models were additionally adjusted for energy intake; ^2^ Crude model is adjusted for child’s sex and age at FFQ; ^3^ Covariate-adjusted model additionally includes maternal cardiometabolic health, age, BMI, smoking, alcohol intake and folic acid supplementation during pregnancy, pregnancy complications, household income, parental education, child’s birth weight, breastfeeding, timing of introduction of fruit and vegetables, receiving dietary supplements, glycemic load, diet quality score, physical activity, screen time, and smoking in the household; ^4^ In our study population, a 0.026 SDS higher HDL-C corresponds to approximately 0.008 mmol/L or 0.31 mg/dL; and a 0.020 SDS lower triglyceride concentrations to approximately 0.010 mmol/L or 0.89 mg/dL; * *p*-value < 0.05. Abbreviations: BF%, body fat percentage; DBP, diastolic blood pressure; DF, dietary fiber; HDL-C, high-density lipoprotein cholesterol; SBP, systolic blood pressure; SDS, standard deviation score.

**Table 3:** Covariate-adjusted associations between DF intake and energy-adjusted DF intake from cereals, from potatoes, from fruits and vegetables, and from legumes (per 1 g/day) and cardiometabolic outcomes.

	Cereals (per 1 g/day)		Potatoes (per 1 g/day)		Fruit & Vegetables (per 1 g/day)		Legumes (per 1 g/day)	
	DF Intake	Energy-Adjusted DF Intake ^1^	DF Intake	Energy-Adjusted DF Intake ^1^	DF Intake ^1^	Energy-Adjusted DF Intake ^1^	DF Intake	Energy-Adjusted DF Intake ^1^
Cardiometabolic risk factor score (SDS)	−0.006	−0.004	−0.050 *	−0.051 *	−0.009	−0.009	−0.032	−0.03
*n* = 1311	(−0.026, 0.014)	(−0.026, 0.019)	(−0.093, −0.008)	(−0.096, −0.009)	(−0.034, 0.017)	(−0.034, 0.016)	(−0.111, 0.048)	(−0.110, 0.049)
BF% (SDS)	−0.009	−0.005	0	−0.001	0.006	0.006	−0.001	0
*n* = 1984	(−0.024, 0.005)	(−0.022, 0.011)	(−0.032, 0.032)	(−0.033, 0.032)	(−0.012, 0.025)	(−0.013, 0.025)	(−0.061, 0.059)	(−0.059, 0.060)
HDL-C (SDS)	0.008	0.002	0.028 *	0.033	0.027 *	0.028 *	0.035	0.033
*n* = 1383	(−0.013, 0.029)	(−0.021, 0.024)	(0.016, 0.072)	(−0.012, 0.077)	(0.000, 0.054)	(0.002, 0.054)	(−0.047, 0.117)	(−0.048, 0.115)
Insulin (SDS)	0.001	−0.008	−0.018	−0.01	0.012	0.013	−0.015	−0.016
*n* = 1378	(−0.019, 0.022)	(−0.030, 0.015)	(−0.055, 0.033)	(−0.053, 0.034)	(−0.015, 0.039)	(−0.014, 0.040)	(−0.097, 0.067)	(−0.098, 0.066)
Triglycerides (SDS)	0.011	−0.007	−0.075 *	−0.076 *	−0.02	−0.021	−0.015	−0.014
*n* = 1381	(−0.010, 0.032)	(−0.015, 0.000)	(−0.120, −0.030)	(−0.121, −0.032)	(−0.048, 0.007)	(−0.050, 0.006)	(−0.098, 0.068)	(−0.098, 0.069)
DBP (SDS)	−0.007	−0.003	−0.001	−0.0034	−0.004	−0.005	−0.016	0.017
*n* = 1939	(−0.024, 0.009)	(−0.021, 0.016)	(−0.038, 0.035)	(−0.040, 0.033)	(−0.025, 0.017)	(−0.026, 0.016)	(−0.052, 0.084)	(−0.051, 0.085)
SBP (SDS)	−0.009	−0.012	0.004	0.004	0.001	0.002	−0.008	−0.008
*n* = 1939	(−0.026, 0.009)	(−0.032, 0.008)	(−0.034, 0.041)	(−0.033, 0.042)	(−0.021, 0.023)	(−0.021, 0.024)	(−0.028, 0.012)	(−0.028, 0.012)

Values are based on multivariable linear regression models and reflect differences (95% confidence intervals) in individual cardiometabolic outcomes and in cardiometabolic risk factor score (age- and sex-adjusted SD scores) per 1 g/day increase in DF intake from different sources. All models are adjusted for maternal cardiometabolic health, age, BMI, smoking, alcohol intake and folic acid supplementation during pregnancy, pregnancy complications, household income, parental education, child’s birth weight, sex, breastfeeding, timing of introduction of fruit and vegetables, age at food-frequency questionnaire (FFQ), receiving dietary supplements, glycemic load, diet quality score, physical activity, screen time, and smoking in the household. ^1^ DF was analyzed as energy-adjusted DF using the residual method and models were additionally adjusted for energy intake. * *p*-value < 0.05. Abbreviations: BF%, body fat percentage; DBP, diastolic blood pressure; DF, dietary fiber; HDL-C, high-density lipoprotein cholesterol; SBP, systolic blood pressure; SDS, standard deviation score.

## Supplementary Materials

The following are available online at http://www.mdpi.com/2072-6643/8/9/531/s1, Table S1: Population characteristics based on unimputed and imputed data; Table S2: Crude associations between DF intake and cardiometabolic outcomes; Table S3: Crude and covariate-adjusted associations between DF intake and cardiometabolic outcomes among children with complete data on cardiometabolic health (*n* = 1314); Table S4: Sensitivity analyses: associations between DF intake and the cardiometabolic risk factor score without TG or HDL-C; Table S5: Descriptives of children with and without blood samples available.

## Abbreviations

The following abbreviations have been used in the text:

BF%	Body Fat Percentage
BMI	Body Mass Index
DBP	Diastolic Blood Pressure
DF	Dietary Fiber
FFQ	Food Frequency Questionnaire
HDL-C	High Density Lipoprotein Cholesterol
LDL-C	Low Density Lipoprotein Cholesterol
SBP	Systolic Blood Pressure
SDS	Standard Deviation Score
