# Supplementary material for: Associations between Dietary Fiber Intake in Infancy and Cardiometabolic Health at School Age: The Generation R Study
Source: Nutrients. 2016 Aug 30;8(9):531. doi: 10.3390/nu8090531 (PMC5037518; doi:10.3390/nu8090531)
Supplement: Supplementary file 1 [file nutrients-08-00531-s001.docx]

Supplementary Materials: Associations between Dietary Fiber Intake in Infancy and Cardiometabolic Health at School Age: The Generation R Study

Rafaëlle M. A. van Gijssel, Kim V. E. Braun, Jessica C. Kiefte-de Jong, Vincent W.V. Jaddoe,
Oscar H. Franco and Trudy Voortman

**Table S1.** Population characteristics based on unimputed and imputed data.

|  | **Unimputed Data** | **Imputed Data** |
| --- | --- | --- |
|  | **Mean ± SD, median (IQR), or *n* (%)** | **Mean ± SD, median (IQR), or *n* (%)** |
| **Infancy Characteristics** |  |  |
| Gestational age at delivery (weeks)   - Missing | 40.3 (39.1–41.0) | 40.1 (39.3–41.1) |
|  | 2 (0.1) | - |
| Birth weight (g)   - Missing | 3499 (563) | 3499 (563) |
|  | 1 (0.1) | - |
| Sex (boys) | 1001 (49.3) | NI |
| Receiving breastfeeding   - Never - Partial in the first 4 months - Exclusively in the first 4 months - Missing |  |  |
|  | 179 (8.8) | 272 (13.3) |
|  | 1107 (54.5) | 1154 (56.8) |
|  | 548 (27.0) | 606 (29.9) |
|  | 198 (9.7) | - |
| Timing of introduction of fruit and vegetables foods   - <4 months - 4–6 months - >6 months - Missing |  |  |
|  | 109 (5.4) | 162 (7.9) |
|  | 1479 (72.8) | 1716 (84.4) |
|  | 89 (4.4) | 154 (7.7) |
|  | 355 (17.4) | - |
| **Characteristics at Nutritional Assessment at 1 Year** |  |  |
| Age (months) | 12.9 (12.6–13.9) | NI |
| Dietary fiber intake (g/day)   - DF from cereals - DF from potatoes - DF from fruit and vegetables - DF from legumes | 15.0 (4.3) | NI |
|  | 8.0 (6.2–10.0) | NI |
|  | 1.1 (0.4–1.9) | NI |
|  | 4.7 (3.2–6.2) | NI |
|  | 0.2 (0.0–0.6) | NI |
| Dietary fiber intake compared with guideline   - Below 15.0 g/day - Equal and above 15.0 g/day |  |  |
|  | 1086 (53.4) | NI |
|  | 946 (46.6) | NI |
| Energy intake (kcal/day) | 1267 (1070–1491) | NI |
| Glycemic load | 86.9 (73.2–105.7) | NI |
| Receiving food supplements at 1 year | 973 (47.9) | NI |
| **Characteristics at Cardiometabolic Health Assessment at 6 Years** |  |  |
| Age (year) | 5.93 (5.84–6.05) | NI |
| Height (cm) | 118 (115–122) | NI |
| Weight (kg) | 21.8 (20.2–23.8) | NI |
| BMI (kg/m^2^) (*n =* 1995) | 15.6 (15.0–16.5) | NI |
| BF% (*n =* 1988) | 23.1 (20.4–26.4) | NI |
| Serum HDL-C (mmol/L) (*n* = 1385) | 1.33 (0.30) | NI |
| Serum Insulin (pmol/L) (*n* = 1380) | 114 (63.8–183.6) | NI |
| Serum Triglycerides (mmol/L) (*n* = 1383) | 0.98 (0.72–1.29) | NI |
| Diastolic blood pressure (mmHg) (*n* = 1943) | 60 (6) | NI |
| Systolic blood pressure (mmHg)(*n* = 1943) | 102 (8) | NI |
| Physical activity (h/day)   - Missing | 1.57 (1.00–2.39) | 1.60 (1.00–2.43) |
|  | 391 (19.2) | - |
| Screen time (h/day)   - Missing | 1.14 (0.75–1.71) | 1.14 (0.75–1.71) |
|  | 292 (14.3) | - |
| Passive smoking in household   - Seldom or never - Less than once a week - More than once a week - Missing |  |  |
|  | 1652 (81.3) | 1829 (90.0) |
|  | 41 (2.0) | 71 (3.5) |
|  | 105 (5.2) | 132 (6.5) |
|  | 234 (11.5) | - |
| **Parental Characteristics** |  |  |
| Maternal at enrolment (year)   - Missing | 32.3 (29.7–34.6) | 32.3 (29.7–34.6) |
|  | 177 (8.7) | - |
| Maternal BMI at enrolment (kg/m^2^)   - Missing | 23.3 (21.6–25.7) | 23.3 (21.7–25.8) |
|  | 177 (8.7) | - |
| Household income per month   - <€2,200 - ≥€2,200 - Missing |  |  |
|  | 358 (17.7) | 446 (22.0) |
|  | 1419 (69.8) | 1586 (78.0) |
|  | 255 (12.5) | - |
| Educational level parents   - No higher education - One parent higher education - Both parents higher education - Missing |  |  |
|  | 345 (18.6) | 421 (20.7) |
|  | 441 (23.8) | 495 (24.4) |
|  | 1066 (52.5) | 1116 (54.9) |
|  | 180 (8.9) | - |
| Mothers’ prepregnancy cardiometabolic health   - Hypercholesterolemia, diabetes mellitus, or hypertension - No comorbidities - Missing |  |  |
|  | 1411 (69.4) | 1939 (95.4) |
|  | 43 (2.1) | 93 (4.6) |
|  | 578 (28.5) | - |
| Smoking during pregnancy   - Never - Until pregnancy was known - Continued - Missing |  |  |
|  | 1461 (71.9) | 1606 (79.0) |
|  | 191 (9.4) | 213 (10.5) |
|  | 192 (9.4) | 213 (10.5) |
|  | 188 (9.3) | - |
| Alcohol consumption during pregnancy   - Never - Until pregnancy was known - Continued - Missing |  |  |
|  | 520 (25.6) | 627 (30.9) |
|  | 278 (13.7) | 364 (17.9) |
|  | 880 (43.3) | 1041 (51.2) |
|  | 354 (17.4) | - |
| Use of folic acid supplements during pregnancy   - Start periconceptional - Start in first 10 weeks of pregnancy - No - Missing |  |  |
|  | 968 (47.6) | 1245 (61.3) |
|  | 450 (22.1) | 599 (29.4) |
|  | 111 (5.5) | 188 (9.3) |
|  | 503 (24.8) | - |
| Pregnancy complications   - Gestational hypertension, preeclampsia, or gestational  diabetes mellitus - No complications - Missing |  |  |
|  | 133 (6.5) | 183 (9.1) |
|  | 1662 (81.8) | 1849 (90.9) |
|  | 237 (11.7) | - |

Abbreviations: DF, dietary fiber; HDL, high density lipoprotein; IQR, interquartile range.

**Table S2.** Crude associations between DF intake from different sources and cardiometabolic outcomes.

| **Cardiometabolic Outcome** | **DF from Cereals (per 1 g/day)** | | **DF from Potatoes (per 1 g/day)** | | **DF from Fruits and Vegetables (per 1 g/day)** | | **DF from Legumes (per 1 g/day)** | |
| --- | --- | --- | --- | --- | --- | --- | --- | --- |
|  | **DF Intake** ^1^ | **Energy-Adjusted DF Intake** ^1,2^ | **DF Intake** ^1^ | **Energy-Adjusted DF Intake** ^1,2^ | **DF Intake** ^1^ | **Energy-Adjusted DF Intake** ^1,2^ | **DF Intake** ^1^ | **Energy-Adjusted DF Intake** ^1,2^ |
| Cardiometabolic risk factor score | −0.016 | −0.007 | −0.039 | −0.043 * | −0.01 | −0.01 | −0.016 | −0.019 |
| *n* = 1311 | (−0.016, 0.003) | (−0.028, 0.014) | (−0.078, 0.001) | (−0.081, −0.005) | (−0.033, 0.013) | (−0.035, 0.014) | (−0.095, 0.062) | (−0.097, 0.060) |
| BF% (SDS) | −0.012 | −0.01 | −0.007 | 0.012 | −0.003 | −0.001 | 0.01 | 0.01 |
| *n* = 1984 | (−0.026, 0.025) | (−0.027, −0.006) | (−0.024, 0.037) | (−0.020, 0.043) | (−0.020, 0.015) | (−0.019, 0.018) | (−0.050, 0.070) | (−0.051, 0.072) |
| HDL-C (SDS) | 0.011 | 0.007 | 0.028 | 0.003 | 0.02 | 0.018 | 0.031 | 0.039 |
| *n* = 1383 | (0.002, 0.021) | (−0.016, 0.029) | (−0.009, 0.065) | (−0.015, 0.066) | (−0.002, 0.042) | (−0.007, 0.042) | (−0.044, 0.116) | (−0.041, 0.119) |
| Insulin (SDS) | 0 | −0.007 | −0.012 | −0.014 | 0.01 | 0.007 | −0.02 | −0.001 |
| *n* = 1378 | (−0.010, 0.009) | (−0.029, 0.015) | (−0.053, 0.028) | (−0.065, 0.023) | (−0.013, 0.034) | (−0.018, 0.033) | (−0.100, 0.060) | (−0.094, 0.067) |
| Triglycerides (SDS) | 0.012 | 0.018 | −0.054 * | −0.061 * | −0.011 | −0.013 | −0.005 | −0.014 |
| *n* = 1381 | (0.002, 0.021) | (−0.005, 0.041) | (−0.095, −0.013) | (−0.103, −0.018) | (−0.035, 0.013) | (−0.039, 0.012) | (−0.085, 0.076) | (−0.096, 0.067) |
| DBP (SDS) | −0.008 | −0.004 | 0.001 | 0.006 | −0.01 | −0.007 | 0.025 | 0.026 |
| *n* = 1939 | (−0.015, 0.000) | (−0.013, 0.005) | (−0.016, 0.018) | (−0.012, 0.023) | (−0.028, 0.009) | (−0.027, 0.013) | (−0.043, 0.093) | (−0.028, 0.080) |
| SBP (SDS) | −0.006 | −0.014 | 0.012 | 0.007 | −0.001 | 0 | −0.006 | −0.01 |
| *n* = 1939 | (−0.014, 0.002) | (−0.023, −0.004) | (−0.023, 0.046) | (−0.025, 0.038) | (−0.020, 0.019) | (−0.025, 0.016) | (−0.023, 0.012) | (−0.028, 0.009) |

Values are based on multivariable linear regression models and reflect differences (95% CI) in individual cardiometabolic outcomes and in cardiometabolic risk factor score (age- and sex-adjusted SD scores) per 1 g/day increase in DF intake from different sources. DF intake from different sources were analyzed in separate models. ^1^ Crude model is adjusted for child’s sex and age at FFQ; ^2^ DF was analyzed as energy-adjusted DF using the residual method and is additional adjusted for energy intake. * *p*-value < 0.05. Abbreviations: BF%, body fat percentage; DBP, diastolic blood pressure; DF, dietary fiber; HDL-C, high-density lipoprotein cholesterol; SBP, systolic blood pressure; SDS, standard deviation score.

**Table S3.** Crude and covariate-adjusted associations between DF intake and cardiometabolic outcomes among children with complete data on cardiometabolic health (*n* = 1314).

| **Cardiometabolic Outcome** | **DF Intake (per 1 g/day)** | | **Energy-Adjusted DF Intake (per 1 g/day)** | |
| --- | --- | --- | --- | --- |
|  | **Crude Model ^1^** | **Covariate-Adjusted Model ^2,3^** | **Crude Model ^1^** | **Covariate-Adjusted Model ^2,4^** |
| Cardiometabolic risk factor score | −0.014 * | −0.022 * | −0.023 * | −0.022 * |
|  | (−0.024, −0.003) | (−0.037, −0.006) | (−0.038, −0.007) | (−0.038, −0.006) |
| BF% (SDS) | −0.005 | −0.004 | −0.004 | −0.001 |
|  | (−0.011, 0.001) | (−0.018, 0.009) | (−0.012, 0.003) | (−0.015, 0.013) |
| HDL-C (SDS) | 0.011 * | 0.027 * | 0.033 * | 0.025 * |
|  | (0.012, 0.024) | (0.011, 0.044) | (0.005, 0.038) | (0.008, 0.042) |
| Insulin (SDS) | 0.001 | −0.004 | −0.006 | −0.001 |
|  | (−0.005, 0.007) | (−0.013, 0.020) | (−0.014, 0.003) | (−0.018, 0.016) |
| Triglycerides (SDS) | −0.008 | −0.022 * | −0.017 * | −0.022 * |
|  | (−0.021, 0.004) | (−0.038, −0.005) | (−0.033, −0.001) | (−0.039, −0.004) |
| DBP (SDS) | −0.005 | −0.004 | −0.004 | −0.003 |
|  | (−0.010, 0.001) | (−0.020, 0.011) | (−0.012, 0.011) | (−0.019, 0.013) |
| SBP (SDS) | −0.004 | −0.006 | −0.011 | −0.008 |
|  | (−0.010, 0.002) | (−0.022, 0.010) | (−0.027, 0.004) | (−0.025, 0.008) |

Values are based on multivariable linear regression models and reflect differences (95% CI) in individual cardiometabolic outcomes and in cardiometabolic risk factor score (age- and sex-adjusted SD scores) per 1 g/day increase in DF intake and energy-adjusted DF intake. ^1^ Crude model is adjusted for child’s sex and age at FFQ; ^2^ Covariate-adjusted model includes additional mother’s cardiometabolic health, maternal age, maternal BMI, household income, educational level of the parents, smoking, alcohol intake and folic acid supplementation during the pregnancy, pregnancy complications, child’s birth weight, receiving breastfeeding at 4 months, timing of introduction of fruit and vegetables, receiving food supplements, physical activity, screen time, smoking in the household, and glycemic load; ^3^ DF was analyzed as glycemic load-adjusted DF using the residual method; ^4^ DF was analyzed as energy- and glycemic load-adjusted DF using the residual method is additional adjusted for kcal intake per day. * *p*-value < 0.05. Abbreviations: BF%, body fat percentage; DBP, diastolic blood pressure; DF, dietary fiber; HDL-C, high-density lipoprotein cholesterol; SBP, systolic blood pressure; SDS, standard deviation score.

**Table S4.** Sensitivity analyses: associations between DF intake and the cardiometabolic risk factor score without TG or HDL-C.

|  | **DF Intake (per 1 g/day)** | | **Energy-Adjusted DF Intake (per 1 g/day) ^3^** | |
| --- | --- | --- | --- | --- |
|  | **Crude Model ^1^** | **Covariate-Adjusted Model** | **Crude Model ^1^** | **Covariate-Adjusted Model ^2^** |
| Cardiometabolic risk factor score excluding TG  *n* = 1314 | −0.014 * | −0.017 * | −0.021 * | −0.017 * |
|  | (−0.019, −0.008) | (−0.031, −0.002) | (−0.035, −0.006) | (−0.033, −0.002) |
| Cardiometabolic risk factor score excluding HDL  *n* = 1314 | −0.008 | −0.013 | −0.017 * | −0.015 * |
|  | (−0.017, 0.002) | (−0.027, 0.001) | (−0.032, −0.002) | (−0.030, −0.000) |

Values are based on multivariable linear regression models and reflect differences (95% CI) in cardiometabolic risk factor score (SDS) per 1 g/day increase in DF intake and energy-adjusted DF intake. ^1^ Crude model is adjusted for child’s sex and age at FFQ; ^2^ Covariate-adjusted model is additionally adjusted for maternal age, maternal BMI, household income, educational level of the parents, smoking, alcohol intake and folic acid supplementation during the pregnancy, pregnancy complications, child’s birth weight, receiving breastfeeding at 4 months, timing of introduction of fruit and vegetables, receiving food supplements, physical activity, screen time, smoking in the household, and glycemic load; ^3^ The models for energy-adjusted DF are additionally adjusted for energy intake. * *p*-value < 0.05. Abbreviations: BF%, body fat percentage; DBP, diastolic blood pressure; DF, dietary fiber; HDL-C, high-density lipoprotein cholesterol; SBP, systolic blood pressure; SDS, standard deviation score.

**Table S5.** Descriptives of children with and without blood samples available.

|  | **With Blood Samples** | **Without Blood Samples** | ***p*-Value ^1^** |
| --- | --- | --- | --- |
|  | **Mean (SD)** | **Mean (SD)** |  |
| DF intake (g/day) | *n* = 1389 | *n* = 643 | 0.563 |
|  | 15.1 (4.28) | 14.9 (4.15) |  |
| - DF intake from  cereals (g/day) | *n* = 1389 | *n* = 643 | 0.703 |
|  | 8.15 (2.76) | 8.22 (2.77) |  |
| - DF intake from potatoes (g/day) | *n* = 1389 | *n* = 643 | 0.495 |
|  | 1.44 (1.52) | 1.42 (1.42) |  |
| - DF intake from fruits and vegetables (g/day) | *n* = 1389 | *n* = 643 | 0.604 |
|  | 5.02 (2.33) | 4.78 (2.23) |  |
| - DF intake from legumes (g/day) | *n* = 1389 | *n* = 643 | 0.945 |
|  | 0.47 (0.70) | 0.44 (0.71) |  |
| BF% | *n* = 1364 | *n* = 624 | 0.784 |
|  | 23.4 (4.45) | 23.9 (4.45) |  |
| DBP (mmHg) | *n* = 1341 | *n* = 602 | 0.252 |
|  | 60 (6.21) | 61 (6.43) |  |
| SBP (mmHg) | *n* = 1341 | *n* = 602 | 0.131 |
|  | 101 (7.64) | 102 (8.17) |  |

^1^ Values are based on independent *t*-tests for differences in characteristics between children with and without blood samples available. Abbreviations: BF%, body fat percentage; DBP, diastolic blood pressure; DF, dietary fiber; g/day, gram per day; mmHg, millimeter of mercury; *n*, number of population; SBP, systolic blood pressure.
